# Disruption of the Serotonergic System after Neonatal Hypoxia-Ischemia in a Rodent Model

**DOI:** 10.1155/2012/650382

**Published:** 2012-02-08

**Authors:** Kathryn M. Buller, Julie A. Wixey, Hanna E. Reinebrant

**Affiliations:** ^1^Royal Brisbane and Women's Hospital, The University of Queensland, Herston, QLD 4029, Australia; ^2^Clinical Neuroscience, Perinatal Research Centre, The University of Queensland Centre for Clinical Research, Royal Brisbane and Women's Hospital, The University of Queensland, Herston, QLD 4029, Australia

## Abstract

Identifying which specific neuronal phenotypes are vulnerable to neonatal hypoxia-ischemia, where in the brain they are damaged, and the mechanisms that produce neuronal losses are critical to determine the anatomical substrates responsible for neurological impairments in hypoxic-ischemic brain-injured neonates. Here we describe our current work investigating how the serotonergic network in the brain is disrupted in a rodent model of preterm hypoxia-ischemia. One week after postnatal day 3 hypoxia-ischemia, losses of serotonergic raphé neurons, reductions in serotonin levels in the brain, and reduced serotonin transporter expression are evident. These changes can be prevented using two anti-inflammatory interventions; the postinsult administration of minocycline or ibuprofen. However, each drug has its own limitations and benefits for use in neonates to stem damage to the serotonergic network after hypoxia-ischemia. By understanding the fundamental mechanisms underpinning hypoxia-ischemia-induced serotonergic damage we will hopefully move closer to developing a successful clinical intervention to treat neonatal brain injury.

## 1. General Characteristics of Neonatal Brain Injury

Approximately 4 in 1000 babies are born each year with brain damage. Being born premature (<37 weeks gestation) and exposure to a hypoxic-ischemic insult (HI; reduced oxygen and blood flow to the brain) are the major risk factors that contribute to this statistic [1, 2]. An HI insult can ensue after many possible factors including placental dysfunction, haemorrhage, hypotension, umbilical cord occlusion, and stroke [1]. A considerable number of these preterm neonates estimate as high as 50% [3], develop neurological and functional impairments such as cerebral palsy, motor deficits, sleep disorders, hyperactivity, anxiety, depression, and cognitive and autonomic disabilities [4–8]. These lifelong disabilities place enormous burdens on the individual as well as family, healthcare, educational, and community resources. 

Although significant advances in neonatal care have increased survival rates of preterm infants, particularly those less than 28 weeks gestation, a concomitant decrease in morbidity has not been achieved. In addition, aside from the recent development of early cooling of the neonatal brain [9, 10], there is no therapeutic intervention available to treat neonatal brain injury. Thus the substantial associated life-long burdens are growing and there is an urgent need to identify neuroprotective drugs that target neuronal networks to prevent, slow, or abate the deleterious effects of HI in the neonatal brain. 

White matter damage is a hallmark feature of brain injury after HI in the preterm neonate. Enlarged ventricles (ventriculomegaly), loss of vulnerable oligodendrocyte progenitor cells, periventricular leukomalacia (PVL), hypomyelination, thinning of the corpus callosum, astrogliosis, and microgliosis are typical features of white matter damage [11–16]. Characterising white matter injury and searching for the mechanisms contributing to this injury have been major avenues of investigation in the area of preterm HI brain injury. However, neuronal loss is also a critical neuropathological feature of HI and the pattern of brain injury in preterm neonates is described as a combination of white and grey matter damage [11–13]. Moreover, it is plausible that disrupted neuronal function and neural circuit connectivity are a consequence of white matter loss and axonal disruption.

## 2. Neuronal Damage in the Preterm HI Brain

With the advent of more sophisticated and higher resolution imaging techniques scientists are beginning to discriminate white and gray matter, delineate neural connectivity, and identify biochemical markers so that brain injury in the neonate is increasingly being characterized in much finer detail. It is well established that there are volumetric reductions in certain brain areas of HI-affected preterm infants including the thalamus, basal ganglia, and cerebral cortex and that these effects are manifested in association with PVL and other white matter features [17–21]. Axonal pathology and neuronal injury have been reported in these regions as well as in the brainstem, cerebellum, striatum, hippocampus, and hypothalamus after HI in the human preterm brain [8, 22–24] and animal models [25–28]. Furthermore, long-term changes in neuronal neurotransmitter content and release can also occur after neonatal HI [29–32]. Disruption of neuropeptides and neurotransmitters, critical for the development of synapses and formation of neuronal networks, has been postulated to underlie behavioural deficits and neuroendocrine disorders in the growing child and adult human with a history of preterm HI [33]. It is pertinent that some types of neurons (e.g., dopaminergic, noradrenergic, and cholinergic neurons) may be more vulnerable to perinatal injury than others (e.g., magnocellular neurons in the hypothalamus) [28, 34–36]. 

Identifying which specific neuronal phenotypes are vulnerable to HI, where in the brain they are damaged, the timing and mechanisms underlying neuronal losses are necessary directions to establish the anatomical substrates underpinning functional impairments in HI-affected neonates. These are important issues to determine because if particular neuronal phenotypes or brain regions are injured at different times or differ in their vulnerability to HI then selective neuroprotective interventions may also be temporally and spatially distinct. One neural network that we have a particular focus on is the serotonergic system in the brain. 

## 3. The Serotonergic System: A Candidate Network Disrupted after Neonatal HI

Virtually all brain regions reportedly injured after neonatal HI receive substantial serotonergic fibre projections from the brainstem. In addition, the rostral brainstem, where serotonergic cell bodies reside, is damaged after neonatal HI [8]. It is well established that interruption of the central serotonergic system can lead to numerous functional deficits and many outcomes are similar to those observed in preterm neonates exposed to HI. These observations prompted us to hypothesise the serotonergic network in the brain is a major system that is disrupted after preterm HI and that this system is a pivotal neural candidate to target with neuroprotective interventions after preterm HI. 

Serotonin (5-hydroxytryptamine, 5-HT) is pivotal in fetal and postnatal brain development [37]. The serotonergic network in the brain develops very early during gestation and is one of the first transmitter systems to appear in the developing brain. Indicative of its pervasive innervation of the central nervous system in the postnatal and mature brain, 5-HT is a neurochemical that is involved in a vast array of functions. In addition, dysfunction of serotonergic neurotransmission has been implicated in a host of physiological, metabolic, and behavioural changes in disease states such as epilepsy, depression, movement disorders, autism, anxiety and sudden infant death syndrome (SIDS) [38–43]. In the context of neonatal brain injury, it is pertinent that many of these deficits match those observed in HI-affected neonates [4, 5, 7, 44]. In addition, decreased serotonergic function is a hallmark feature of depression and depressed patients show 31% loss of dorsal raphé neurons [45]. Cerebral palsy is a notable disability in some HI-affected neonates and these patients have been reported to suffer depression [46, 47]. Although, whether altered serotonergic function accounts for certain HI-induced neurological deficits is not known. It is important to first characterise the effects of neonatal HI on major elements of the serotonergic system in the brain and begin to decipher whether these specific nuclei constitute primary candidate networks that underpin neonatal HI-induced neurological deficits.

Utilising a postnatal day 3 (P3) HI model of preterm HI we have recently investigated how P3 HI affects the serotonergic system in the brain. The P3 rat pup is subjected to HI by right common carotid artery ligation followed by 6% oxygen for 30 min. In the rat, the P3 brain development stage is analogous to the preterm human neonate brain at approximately 24–28 weeks gestation in terms of cellular development, number of synapses, neurochemical development, and cortical organization [48]. This preclinical model produces typical behavioural and pathological features including encephalopathy and hypomyelination observed in human preterm neonates affected by HI [4, 28, 48–51].

## 4. The Synthesis and Release of 5-HT in the Central Nervous System

**Figure 1:**
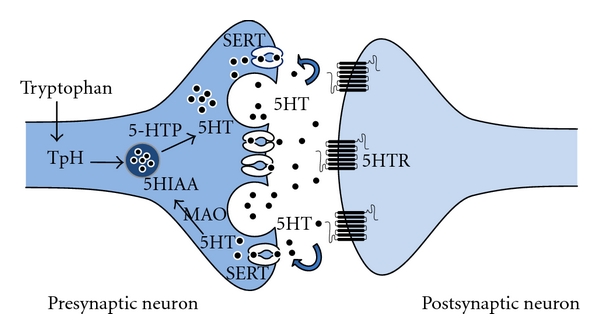
Schematic diagram depicting the major pathways involved in the synthesis, release, re-uptake and metabolism of serotonin in serotonergic neurons. Components of the figure have been modified from Motifolio. TpH: tryptophan hydroxylase; 5-HTP: 5-hydroxy-L-tryptophan; 5-HT: serotonin; SERT: serotonin transporter; MAO: monoamine oxidase; 5-HIAA: 5-hydroxyindoleacetic acid; 5-HTR: serotonergic receptor.

Serotonin is synthesised in the brain in serotonergic neurons from the amino acid L-tryptophan and its metabolite 5-hydroxytryptophan (5-HTP). Synthesis occurs via tryptophan hydroxylase (TpH), 5-HT's rate-limiting enzyme and a second enzyme amino acid decarboxylase. Two isoforms of TpH are known to exist (TpH_1_ and TpH_2_) but only TpH_2_ is found in the brain [52]. The major regulator of 5-HT levels in the brain is the serotonin transporter (SERT). The transporter consists of 12 transmembrane domains that span the presynaptic membrane of 5-HT-releasing cells [53]. Localised on the presynaptic membrane of serotonergic neurons, SERT terminates serotonergic signalling by the efficient reuptake of extracellular serotonin back into the presynaptic neuron (Figure 1) thereby controlling the duration of action and post-synaptic signalling of 5-HT in the brain. Consequently, SERT is a major target for drugs such as selective serotonin reuptake inhibitors (SSRIs) that can increase 5-HT availability in the brain and are useful drugs in the treatment of depression. Serotonin is also broken down by monoamine oxidase (MAO) enzymes, preferentially MAO-A, into 5-hydroxyindoleacetic acid (5-HIAA); serotonin's major metabolite. 

Nine groups of 5-HT-containing cell bodies represented in raphé subdivisions in the pons and upper brainstem were first described using histochemical techniques and designated B_1−9_ [54]. The bilateral raphé subdivisions are predominantly populated with serotonergic neuronal cell bodies and provide an extensive serotonergic network throughout the central nervous system. Based on their cytoarchitecture, neurochemistry, and neural projections, nomenclature for the clusters of 5-HT neurons describes their location in the dorsal, lateral, midline, or caudal portion of the pons and medulla oblongata [55, 56].

## 5. Serotonergic Damage in the Immature Brain after HI

In human neonates with HI encephalopathy tryptophan hydroxylase, the 5-HT rate-limiting enzyme, is reduced in the brainstem [41, 57]. Damage to human dorsal brainstem nuclei, where serotonergic cell bodies are located, is also apparent [8]. However, until our initial study in 2010 in a rodent P3 HI model [58], information about the effects of HI on specific raphé nuclei was scarce. We found an overall significant loss of 5-HT-positive raphé neurons after P3 HI, consistent with previous animal studies [59, 60] and reports from human neonates [8]. However it is interesting that certain serotonergic raphé nuclei appear to be more vulnerable to P3 HI-induced injury than others. One week after P3 HI, 5-HT-positive neuronal losses occur in the dorsal raphé caudal, dorsal raphé ventrolateral, and dorsal raphé dorsal nuclei. In contrast, the dorsal raphé interfascicular and the raphé magnus nuclei showed no reduction in number of 5-HT-positive neurons on P10 and P45. Six weeks after P3 HI, on P45, only the dorsal raphé ventrolateral and the dorsal raphé dorsal demonstrated a maintained and significant decrease in numbers of 5-HT-positive neurons [58]. 

The rostrocaudal distribution of the raphé serotonergic neurons may determine their vulnerability to HI injury. It is evident that the anterior raphé subdivisions are more affected by P3 HI than the more posterior and caudally located raphé nuclei such as the raphé magnus and the dorsal raphé interfascicular nuclei [58]. The topographical clustering of different raphé subdivisions in the midbrain and brainstem also represents differential connectivity patterns in the brain. As such the dorsal raphé caudal, dorsal raphé ventrolateral, and dorsal raphé dorsal nuclei primarily innervate the cerebral cortex, basal ganglia, thalamus, hypothalamus, hippocampus, and amygdala [55, 61, 62]. In contrast, the more caudal nuclei predominantly send neural projections to the spinal cord and other parts of the brainstem [63]. The afferent and efferent connections of each raphé subdivision are integral to producing characteristic serotonergic-dependent functions. Thus selective losses of serotonergic raphé nuclei may underpin particular deficits reported in HI-affected preterm infants. On the other hand, it appears that the more caudal raphé magnus and dorsal raphé interfascicular nuclei are not susceptible to P3 HI injury and therefore the serotonergic innervation of the spinal cord remains relatively intact and functional after P3 HI [58]. Indeed previous reports suggest that spinal cord injury only occurs after severe neonatal HI insults [64, 65].

Functional disruption of the serotonergic system after neonatal HI is clearly reflected in the reduction in 5-HT levels in the brain. We and others have demonstrated reduced 5-HT levels in cortical, thalamic, and brainstem regions after HI produced in the immature rodent brain [66, 67]. The losses of brainstem dorsal raphé neurons and their neural projections after HI are most likely responsible for the reduced 5-HT levels in the forebrain. Although regional differences are apparent, the association between direct serotonergic neural inputs to forebrain regions from specific raphé nuclei in the brainstem is not known. Thus determining whether specific ascending and descending neural connections are disrupted after HI injury may predict raphé nuclei vulnerability to P3 HI injury and the effects they have on brain regions innervated by serotonergic afferents and efferents. 

In concert with the loss of raphé neurons and reductions in 5-HT levels in the brain, SERT expression is significantly reduced in the brain [58, 67, 68]. We have characterised SERT losses after P3 HI using both Western blot and immunolabelling techniques. The distribution of SERT in fibres, dendrites, cell bodies, and axon terminals [69] makes it an excellent marker of the serotonergic network in the brain [70, 71]. As such the distribution of SERT in the brain closely reflects that of serotonergic neuronal cell bodies and innervating fibres [72, 73]. Serotonergic fibre losses and damage are observed after P3 HI in several key forebrain regions such as the motor and somatosensory cortex, lateral hypothalamus, ventrolateral thalamus, and horizontal limb of the diagonal band [67]. The parallel and concomitant reductions in 5-HT levels and SERT indicate that there was reduced availability of 5-HT for release as well as limited reuptake of 5-HT. This is analogous to findings after ischemia in P7 rat pups whereby there is concurrent attenuation of 5-HT and its major metabolite, 5-hydroxyindoleacetic acid (5-HIAA), suggesting that injury to the serotonergic neuronal network ensues rather than direct modulation of SERT itself or of serotonergic metabolism [66]. We therefore speculate that P3 HI induces disruption to the serotonergic system as a result of loss or damage to serotonergic neurons.

The efficient reuptake of 5-HT is primarily dependent on the localisation of SERT on cell bodies, dendrites, and fibres of serotonergic neurons in the central nervous system [72]. However there is evidence that the reuptake of 5-HT can occur by glial cells whereby SERT may be also localised on astrocytes [74–77] and/or microglial cells [78]. Thus glial SERT could potentially assist in the clearance of 5-HT from the serotonergic synapse [74]. However of the few studies that have specifically examined this possibility, most have only reported localisation of SERT in glial cell lines and primary cultures. Through our own *in vivo* investigations, we have found no evidence of SERT localisation on microglia or astrocytes in the normal or P3 HI-injured neonatal rodent brain (unpublished). 

From our studies it is interesting to note that, in general, proportionately greater serotonergic changes occur in the forebrain regions compared to the brainstem raphé nuclei [58, 67, 68]. This observation has led us to speculate that damage to the serotonergic fibres in the forebrain core/penumbral areas of the HI-injured brain may occur before injury to the brainstem raphé nuclei. In our P3 HI model in the rodent, ligation of the common carotid artery affects a vascular field encompassing primarily forebrain regions, whereas the brainstem lies outside this vascular field and is seemingly spared of immediate hypoxic and ischemic conditions. It has been shown that blood flow to the brainstem tends to increase during HI [79]. In addition we have consistently found that, unlike the forebrain, there is no change in brainstem hemisphere area after P3 HI [28, 58, 67, 68]. The dorsal raphé nuclei can be considered remote from the damaged forebrain sites and therefore serotonergic neuronal injury in the brainstem might develop as a result of secondary mechanisms. One such secondary injury mechanism that we have had a particular focus on is P3 HI-induced neuroinflammation.

## 6. Role of Neuroinflammation in Producing Neuronal Injury

Two phases of injury can be defined after a neonatal HI insult; an early primary phase within 24–48 h causing mainly irreversible injury in the brain and a later secondary injury phase then ensues. Early neuronal injury after HI is thought to evolve primarily via necrosis resulting from excitotoxic damage produced by excessive release of glutamate from presynaptic nerve terminals and astrocytes, causing calcium overload and cell death [80]. Brain injury during the primary phase can also result from high levels of free radicals including reactive nitrogen species and reactive oxygen species accumulating in the brain tissue [81, 82]. Both caspase-dependent and caspase-independent mediators of cell death are also initiated after neonatal HI [83, 84]. 

The subsequent secondary phase can continue for weeks, months, or longer after HI. A vast array of mechanisms may contribute to neuronal injury during this phase and the majority of these have been identified as features of neuroinflammation. Key features of this phase include increased numbers of activated microglia, astrogliosis, increased levels of proinflammatory cytokines (e.g., interleukin-1*β* (IL-1*β*), tumor necrosis factor-*α* (TNF-*α*) and interleukin-6 (IL-6), decreased levels of anti-inflammatory cytokines, increased cyclooxygenase (COX-1 and COX-2) expression, prostaglandins (PGE_2_ and PGI_2_), nuclear factor kappa-light-chain-enhancer of activated B cells, increased expression of chemokines and chemokine receptors, cell adhesion molecule expression, and matrix metalloproteinases [85–89]. Proinflammatory cytokines, particularly IL-1*β* and TNF-*α*, are synthesized and released by activated microglia although IL-1*β* is also expressed by astrocytes and developing oligodendrocytes [90–92]. Astrocytes are important sources of lactate, neurotrophic factors, and pyruvate for neurons and contribute to maintaining neurotransmitter and metabolic homeostasis in the brain [93, 94]. However, although inhibition of astrogliosis after neonatal HI improves the survival of newborn neurons it does not alter infarct volume [95]. The infiltration of peripheral cells such as lymphocytes, neutrophils, and mast cells can also ensue if there is sufficient breakdown and leakage across the blood-brain barrier [89, 96, 97]. The hallmark feature of neuroinflammation in the HI-affected brain that we have focused on, in terms of a potential mechanism underpinning serotonergic neurodegeneration, is the elevated number of activated microglia. 

Numbers of activated microglia peak within the first week after HI although can remain elevated for weeks or months after the initial ischemic episode as observed in human and preclinical studies [68, 84, 89, 91, 98–100]. Microglia are the resident immune cells of the CNS that, in the normal brain, survey the extracellular environment and scavenge and clear the brain of debris and dying cells [101–103]. However microglia can also respond quickly to changes induced by HI in the brain and within 48 h can switch from a resting to an active state, multiply and migrate to sites of ischemic injury [104, 105]. Activated microglia produce and release excessive levels of IL-1*β* and TNF-*α* [50, 84, 89, 100, 106], that are toxic to neurons, can cause neurodegeneration, and negatively affect the neurodevelopment of neonates [107, 108]. We have demonstrated that numbers of activated microglia and levels of TNF-*α* and IL-1*β* in the brain are elevated over the critical first week after P3 HI, particularly in the cortex, thalamic nuclei, and white matter, and closely parallel injury to the serotonergic system [67, 109]. Thus an association between neuroinflammation and serotonergic injury is evident and the period after the P3 HI insult is a critical window of opportunity for interventions that target neuroinflammation. Two anti-inflammatory drugs are proving to be potential interventions to ameliorate HI-induced damage to the serotonergic system. These are minocycline and ibuprofen.

**(a) Effects of Minocycline on the Serotonergic System after Neonatal HI**

The role of activated microglia and raised levels of proinflammatory cytokines in contributing to serotonergic neuronal disruption can be addressed by blocking microglial function with anti-inflammatory drugs. Minocycline is a broad-spectrum antibiotic that also has anti-inflammatory properties in the brain primarily because it is a potent inhibitor of activated microglia [110, 111]. Minocycline does not appear to affect astrocytes after neonatal HI [84, 111, 112]. Minocycline readily crosses the blood-brain barrier after systemic delivery [113, 114] and is an effective neuroprotective intervention when delivered after-insult [50, 67, 83, 115, 116]. The opportunity to alter HI-induced brain injury *after* the insult is an important prospect because clinical diagnosis of HI in the preterm neonate is often not made until 3 days after birth, well into the secondary injury phase. Furthermore it is difficult to predict if an HI insult is imminent and therefore prophylactic treatments during pregnancy or labour are difficult to administer.

Recent studies in the adult rat demonstrate that minocycline reverses 3-nitropropionic acid neurotoxicity-induced changes in 5-HT levels [117] and reduces the 3,4-methylenedioxymethamphetamine-induced reduction in SERT expression [118]. We have now shown in our neonatal model that minocycline, initiated 2 h after P3 HI and administered daily for 1 week, inhibits P3 HI-induced microglial activation and TNF-*α* and IL-1*β* levels and also results in fewer raphé neurons being lost, maintenance of normal 5-HT levels, and increases SERT expression [67]. However not all effects of minocycline on the serotonergic system damage are completely prevented. Furthermore, using the same 1-week-long minocycline regimen, HI-induced neuroinflammation is still inhibited 6 weeks later [68] but minocycline's long-term neuroprotection of the serotonergic system is less effective than at P10. At 6 weeks after-HI SERT expression and serotonergic fibre content appear to be close to control levels but 5-HT levels remain reduced [68]. Nonetheless minocycline, a robust inhibitor of P3 HI-induced neuroinflammatory mediators, significantly improves serotonergic outcomes; however, HI induced damage to the serotonergic network. 

Although minocycline treatment could be a novel therapy to minimise serotonergic changes after neonatal HI and preserve the integrity of 5-HT neurocircuitry in the brain, the use of minocycline in neonates is controversial. Minocycline is an excellent tool to block microglial activation and has considerable neuroprotective effects, not only in neonatal HI animal models. Moreover minocycline has proven to be highly beneficial in numerous adult human trials to treat a variety of neurodegenerative conditions [119–122]. Nevertheless its use in human neonates must be undertaken with caution because of the adverse effects associated with chronic tetracycline that historically have tarnished their administration to neonates [123]. Minocycline can produce bone stunting, staining, and pitting of teeth [123–125]. Tetracyclines may also prevent the binding of bilirubin to albumin and possibly lead to bilirubin-induced brain damage in neonates. In contrast, recent studies have demonstrated that minocycline does not produce some of the side effects historically associated with tetracycline use in neonates [126–129]. The development of new derivatives of minocycline, with fewer adverse side effects, could be promising interventions to develop for clinical translation. Alternatively, given the potential of anti-inflammatory interventions to prevent serotonergic injury, testing other anti-inflammatory drugs that may be more clinically acceptable for use in neonates is a rational approach.

**(b) Effects of Ibuprofen on the Serotonergic System after Neonatal HI**

Nonsteroidal anti-inflammatory drugs (NSAIDs) constitute an alternative anti-inflammatory treatment to stem brain injury after neonatal HI. In this class, drugs such as ibuprofen and indomethacin are commonly used to treat patent ductus arteriosus in preterm neonates [130, 131]. Ibuprofen is a lipophilic compound and after systemic delivery easily crosses the blood-brain barrier [132]. A canonical mechanism of action of NSAIDs is to inhibit cyclooxygenase 1 and 2 enzymes (COX-1, COX-2) and the conversion and synthesis of arachidonic acid to downstream inflammatory effectors such as cytokines and prostaglandins.

Systemic delivery of ibuprofen can inhibit central neuroinflammation and elicit neuroprotective effects although these outcomes have primarily been demonstrated in adult models of cerebral ischemia [133–136]. In human preterm neonates (<28 weeks gestation) indomethacin reduces white matter loss [137] although ibuprofen combined with ascorbic acid treatment in neonates reportedly has little effect on brain injury after severe HI [138]. Consistent with previous studies [86, 139–142], we have shown that COX-2 is elevated in the brain after P3 HI and that ibuprofen significantly prevents this effect as well as P3 HI-induced increases in numbers of activated microglia, IL-1*β*, and TNF-*α* levels [143]. In association with these anti-inflammatory effects ibuprofen ameliorated reductions in cerebral hemisphere size, O4-positive pre-myelinating, O1-positive immature oligodendrocyte cell counts, and myelin content [143]. 

The potential of ibuprofen to be a neuroprotective agent in neonates to stem HI brain injury is further supported by findings that systemic indomethacin or COX-2 inhibitors (NS398) attenuate inflammatory changes as well as functional impairments after neonatal HI in the rodent [142, 144, 145]. In contrast, the effects of NSAIDs on serotonergic neuronal injury after HI are not known. Preliminary evidence in our preclinical HI model indicates that ibuprofen prevents reductions in SERT expression, 5-HT levels (in the frontal cortex and thalamic nuclei), and serotonergic raphé neuronal counts (unpublished). Our findings suggest that ibuprofen is as effective at preventing serotonergic injury however, like minocycline, it does not appear to completely ameliorate damage to this neuronal network. Thus it is plausible that other mechanisms of injury also contribute to HI-induced serotonergic damage in the neonatal brain.

## 7. Lack of P3 HI-Induced Neuroinflammatory Mediators in the Brainstem

From our studies, it is interesting to note that a pattern of neuroinflammation is beginning to emerge. The brainstem dorsal raphé and frontal cortex, for example, represent two areas where neuroinflammatory mediator profiles differ markedly. In the frontal cortex substantial and significant increases in activated microglia and proinflammatory cytokines occur after P3 HI. In contrast, in the brainstem, we have observed that the brainstem does not elicit any major signs of neuroinflammation after P3 HI. Numbers of activated microglia are relatively small, there are no apparent changes in proinflammatory cytokines [67] and more recent data from our laboratory indicates there are no changes in COX-2 expression in raphé serotonergic subdivisions (unpublished). It therefore appears that serotonergic raphé cell bodies are not lost because of local neuroinflammation in the brainstem. Thus, even though inhibition of neuroinflammation has a significant beneficial effect on P3 HI-induced losses of raphé neurons [67], we speculate that the effects of anti-inflammatory drugs such as minocycline are not directly effective at the level of the raphé nuclei. We postulate that the losses of 5-HT-positive neurons in the brainstem after P3 HI, and the neuroprotective effects of minocycline are therefore likely to occur via other, indirect secondary mechanisms.

As stated earlier, at least in our model, the brainstem is located outside the vascular field of the common carotid artery and should not be directly affected by immediate changes in perfusion after HI. Instead neuroinflammation could contribute to brainstem injury via remote actions originating from primary injury sites. Inflammatory mediators may damage afferent and efferent fibres of dorsal raphé nuclei in the forebrain and subsequently compromise the survival of brainstem nuclei by retrograde degeneration and/or target deprivation. The thalamus, substantia nigra, hippocampus, and amygdala have substantial neural connections with primary injury sites (e.g., the cerebral cortex) and can undergo prolonged periods of apoptosis and degeneration in the neonatal brain after HI [25–27, 146]. It has been shown that after ischemic conditions, disrupted somatosensory transmission in the thalamus is associated with increased numbers of thalamic neurons degenerating in the secondary phase [26, 147, 148]. Progressive loss of serotonergic neural connections with damaged areas could lead to the disruption and loss of raphé serotonergic neurons in the brainstem. Indeed the regional differences in vulnerability of 5-HT-positive neurons in the dorsal raphé nuclei after P3 HI [58] might be attributed to the serotonergic innervation pattern to damaged and undamaged forebrain regions. 

The two mechanisms of HI-induced neuroinflammation and neural disruption may not be mutually exclusive. Activated microglia have also been shown to be present in brain regions as a consequence of a loss of connectivity with a target region or axonal interruption [149–151]. It remains to be investigated whether forebrain neuroinflammation after neonatal HI initiates subsequent serotonergic neuronal damage in the remote brainstem via retrograde degeneration and/or target deprivation mechanisms.

## 8. Conclusions and Future Directions

We have identified the serotonergic system as a pervasive network that is disrupted after neonatal HI in a rodent model. The concomitant reductions in SERT, 5-HT levels and 5-HT-positive raphé neurons suggest that serotonergic network injury is a consequence of degenerating serotonergic neurons that project to the HI-damaged forebrain. A change in the levels of 5-HT in the brain gives a “readout” of the functional integrity of the serotonergic system. However determining how the synthesis of 5-HT is affected, the storage, release mechanisms, postsynaptic signaling and the breakdown of 5-HT would further our understanding of how HI-injury affects the serotonergic network and possibly reveal new targets for selective interventions. Moreover key components of the serotonergic system have been a critical focus of our recent work, but whether serotonergic changes manifest as specific impairments of neurological performance is not known. It is plausible that disruption of the serotonergic system may underpin impairments such as hyperactivity, cardiorespiratory, cognitive, and attention deficits observed in preterm children who have experienced neonatal HI [4–7, 152]. Also, current theories implicate a disrupted 5-HT neurocircuitry in the brainstem raphé nuclei as the putative underlying mechanism of cardiorespiratory dysfunction in neonates and increased susceptibility to SIDS [41, 153–155]. Being born preterm is a significant risk factor for SIDS [156] and exposure to a HI insult may be sufficient to alter raphé serotonergic function and increase a neonate's susceptibility to later cardiorespiratory complications and possibly SIDS [41, 155]. 

The serotonergic system does show some degree of recovery weeks after the initial P3 HI insult [58, 68]. Greater density of serotonergic innervation, increased arborization and axonal length, and higher expression of the SERT occur in the postnatal brain; indicating plasticity and temporal differences depending on the region examined [157–159]. It is also remarkable that serotonergic neurons have an ability to sprout and potentially reinnervate after injury [160–162]. In the HI-injured neonatal brain this avenue of investigation remains to be explored, and possibly exploited, to test new therapeutic strategies.

 To date, evidence suggests that both minocycline and ibuprofen are successful postinsult interventions to ameliorate neuroinflammation and reducing neuronal loss. Both of these potential anti-inflammatory treatments could be beneficial for HI-induced injury to other neurons in the brain [163–165]. However neither intervention appears to be sufficient to completely reverse the HI-induced decrease in brain 5-HT levels. The dose, timing, and specificity of anti-inflammatory interventions are likely to be key parameters that dictate their success. Alternatively, selectively targeting the serotonergic system to improve its function, in concert with changes produced by anti-inflammatory drugs, could be an ideal combination treatment to achieve long-term improvement of the serotonergic system after neonatal HI. By understanding the fundamental mechanisms of serotonergic damage after neonatal HI we will hopefully move closer to providing a clinical intervention.
